# Diagnostic Value of Carcinoembryonic Antigen Combined with Multi-Inflammatory Cell Ratios in Colorectal Cancer

**DOI:** 10.1155/2022/4889616

**Published:** 2022-08-26

**Authors:** Xiao-Cui Liu, Yu-Ling Dai, Fei Huang, Zhen-Jie Zhong, Xiao-Fei Liu

**Affiliations:** ^1^Senior Department of Ophthalmology, The Third Medical Center of PLA General Hospital, Beijing 100039, China; ^2^Department of Laboratory Medicine, Weifang Medical University, Weifang, 261053 Shandong Province, China; ^3^Department of Clinical Laboratory, Tengzhou Central People's Hospital, Tengzhou, 277599 Shandong Province, China; ^4^Department of Laboratory Medicine, The 960th Hospital of the PLA Joint Logistics Support Force, Jinan, 250031 Shandong Province, China

## Abstract

**Purpose:**

To evaluate the diagnostic value of carcinoembryonic antigen (CEA) combined with inflammatory cell ratios in colorectal cancer (CRC).

**Methods:**

This retrospective study compared the data of CRC patients with healthy controls. The CEA levels were measured, and the neutrophil-to-lymphocyte ratio (NLR), derived neutrophil-to-lymphocyte ratio (d-NLR), platelet-to-lymphocyte ratio (PLR), and monocyte-to-lymphocyte ratio (MLR) were calculated. The receiver-operating characteristic (ROC) curve was used to assess the diagnostic value of each marker and combined detection. Spearman's rank correlation test was used to analyze the correlation between CEA and NLR, d-NLR, and PLR.

**Results:**

Inflammatory cell ratios and CEA were significantly higher in the CRC group. ROC curve analysis showed that NLR, d-NLR, and PLR had good diagnostic efficacy. The threshold showed that NLR, d-NLR, and PLR were all related to TNM stage, not to age, gender, tumor location, and degree of differentiation. CEA combined with NLR, d-NLR, and PLR (CNDNP) had a significant diagnostic value in CRC. Correlation studies showed that CEA was positively correlated with NLR and d-NLR but not with PLR.

**Conclusion:**

The combination of CEA with CNDNP might be a valuable indicator for CRC diagnosis.

## 1. Introduction

Colorectal cancer (CRC) is one of the most common malignant tumors of the digestive system worldwide, ranking third in morbidity and mortality [1]. Over 1.9 million new CRC cases and 940,000 deaths have been estimated to occur in 2020 worldwide [2]. CRC morbidity and mortality rates are increasing rapidly in many of the highest Human Development Index (HDI) countries; however, they are stabilizing or decreasing in some of the highest HDI countries, and this downward trend is thought to be the result of early diagnosis [3]. At present, the gold criteria for colorectal cancer diagnosis are mainly endoscopic-based pathological examination, but this method has high requirements from both patients and endoscopists and is an invasive and expensive procedure, resulting in poor compliance [4]. In response to the clinical status of high mortality rate and low early diagnosis rate of CRC, there is an urgent need to find sensitive and effective early diagnosis and appropriate prognostic indicators. Currently, serum carcinoembryonic antigen (CEA) has been widely used in the diagnosis and prognosis assessment of CRC and has easy, rapid, and minimal invasion and other advantages, but its sensitivity and specificity are insufficient [5, 6]. Most scholars consider that it is unlikely for a single tumor marker to meet clinical needs and combined detection is required to improve diagnostic efficacy [7–9].

Studies have demonstrated that the tumor microenvironment, which is largely orchestrated by inflammatory cells, is an indispensable participant in the neoplastic process [10, 11]. Neutrophils, monocytes, lymphocytes, and platelets have been shown to be extensively involved in the cancer process [12–15]. Researchers have calculated a number of relevant inflammatory indicators based on routine peripheral blood tests, including neutrophil-to-lymphocyte ratio (NLR), derived neutrophil-to-lymphocyte ratio (d-NLR), platelet-to-lymphocyte ratio (PLR), and monocyte-to-lymphocyte ratio (MLR). Combined detection, such as detection using a combination of CEA and NLR and using a combination of PLR and lymphocyte-to-monocyte ratio (LMR) with CEA, has a better prognostic value [8, 16], but the diagnostic value of more than a three-indicator combination in CRC remains unknown.

In this study, we systematically reviewed the data of 324 CRC patients to investigate the diagnostic value of preoperative CEA combined with inflammatory markers. We explored the diagnostic value of CNDNP, a novel inflammation-based prognostic system with tumor characteristics, in an attempt to provide a reference index for a better diagnosis of CRC.

## 2. Materials and Methods

### 2.1. Study Samples

A total of 324 patients with an initial diagnosis of CRC and without clinical evidence of infection, hematology disease, hyperpyrexia, intestinal perforation, and intestinal obstruction from June 2017 to December 2019 in the 960^th^ Hospital of the PLA Joint Logistics Support Force, China, were retrospectively enrolled in this study. All CRC patients were confirmed in accordance with histological evidence, or patients without histological confirmation were excluded from the present study. The cancer group consisted of 188 male and 136 female cases, aged 32–81 years, with a mean of (61.14 ± 11.51) years.

The enrolled 216 healthy examinees were used as the healthy group. All healthy examinees had no clinical evidence of any diseases according to their health check at the hospital. The healthy group was 126 male and 90 female cases, aged 29-80 years, with a mean of (63.61 ± 12.74) years. There was no significant difference in gender and age between the two groups (*P* > 0.05). This study was approved by the institution ethics commission of the 960^th^ Hospital of the PLA Joint Logistics Support Force, China.

### 2.2. Methods

A 2 mL serum sample was obtained to examine the level of CEA using the electrochemiluminescence method with a Cobas e601 analyzer (Roche, Berlin, Germany). An EDTA-anticoagulated blood sample (2 mL) was collected to detect inflammatory cell counts using a Sysmex XE-2100 automated hematology analyzer (Sysmex Corporation, Kobe, Tokyo, Japan). The NLR, PLR, and MLR were calculated by dividing the absolute number of neutrophils, platelets or monocyte, respectively, by the absolute number of lymphocytes. The d-NLR was calculated using the neutrophil count divided by the result of the white cell count minus neutrophil count.

The average sampling time from CRC patients was 3 days before the date of surgery. All fasting venous blood samples were collected from the enrolled cases and controls between 7 : 00 am and 9 : 00 am and analyzed within 1 h after venipuncture.

### 2.3. Statistical Analysis

Statistical analyses were performed using SPSS 22.0 (Chicago, IL, USA). Continuous variables are presented as the mean ± standard deviation. The normality test was assessed by using the Shapiro-Wilk test. Student's *t*-test and the Mann–Whitney *U* test were used for normally and nonnormally distributed data, respectively. The chi-square test was used to compare intergroup data. The combined detection regression model was built using binary logistic analysis to calculate the overall predictive probability and further evaluated using receiver-operating characteristic (ROC) curve analysis. ROC curve analysis was performed by MedCalc version 9.2 to clarify the diagnostic efficacy of the associated inflammatory markers alone and in combination. When comparing the area under the ROC curve, the statistical method used was proposed by Delong et al. [17]. Correlation analysis was performed using Spearman's rank correlation test. *P* < 0.05 was considered statistically significant.

## 3. Results

### 3.1. Baseline Characteristics

As shown in Table 1, compared with the healthy group, CRC patients had significantly higher levels of CEA, NLR, d-NLR, PLR, and MLR (*P* < 0.01).

### 3.2. ROC Curve Analysis and Determination of the Critical Value

**Figure 1:**
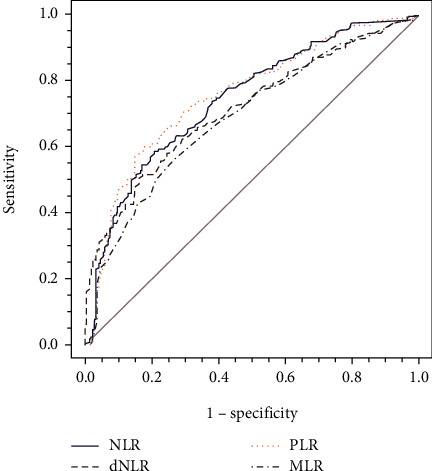
ROC curve analysis of relevant inflammatory indices.

ROC curves were plotted for preliminary clarification of the individual diagnostic efficacy of the relevant inflammatory indices, and the results are shown in Figure 1 and Table 2. The areas under the curve (AUC) of NLR, d-NLR, PLR, and MLR were 0.74 (95% CI 0.70-0.78), 0.71 (95% CI 0.67-0.75), 0.75 (95% CI 0.72-0.79), and 0.68 (95% CI 0.64-0.72), respectively. Table 2 lists the sensitivities and specificities of NLR, d-NLR, PLR, and MLR at their given thresholds. The result showed that PLR had the highest diagnostic efficacy among these inflammatory cell ratios, while MLR had the lowest diagnostic efficacy, and the latter was not used as a diagnostic biomarker for subsequent analysis.

### 3.3. Associations between Clinical Characteristics and NLR, D-NLR, and PLR Levels

To explore the correlation between each marker and clinicopathological features, table statistics were tabulated. As shown in Table 3, the level of the NLR was significantly related to TNM stage (*P* < 0.05), but no significant differences were observed in age, gender, tumor location, and degree of differentiation. Similar results were observed in d-NLR and PLR.

### 3.4. Diagnostic Value of Combined Serum CEA and Routine Blood-Related Inflammatory Indices for Predicting CRC

**Figure 2:**
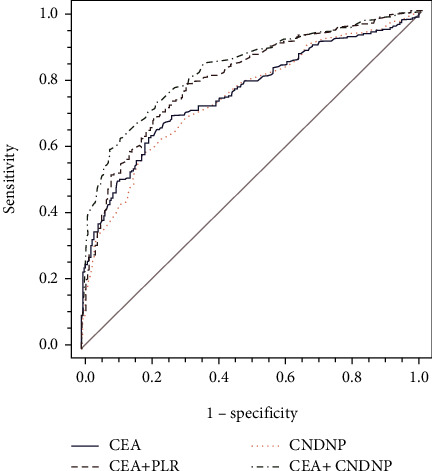
ROC curve analysis of different combined detection schemes.

To obtain higher diagnostic value, we evaluated a combination of NLR, d-NLR, and PLR (CNDNP). As shown in Figure 2 and Table 4, the AUC of CNDNP was 0.74 (95% CI 0.70-0.78), not significant with serum CEA (*Z* = 0.38, *P* > 0.05). However, the highest diagnostic values were found when a combination of CEA with inflammatory markers was used, with CEA + PLR (AUC = 0.80, 95% CI 0.76-0.83) and CEA + CNDNP (AUC = 0.83, 95% CI 0.79-0.86) having the highest diagnostic value, and the difference was statistically significant (*Z* = 2.22, *P* < 0.05). These results indicated that the combined detection could improve the diagnostic efficiency, and that a combination of CEA with CNDNP might be a valuable indicator for CRC diagnosis.

### 3.5. Correlation of CEA and Inflammatory Indices

Correlation analysis was used to preliminarily explore the correlation between CEA and NLR, d-NLR, and PLR. The results showed that CEA was positively correlated with NLR and d-NLR but not with PLR. Although the correlation coefficients were 0.142 and 0.122, respectively, the difference was statistically significant (*P* < 0.05). These results implied that there was a low correlation between CEA and NLR and between CEA and d-NLR.

## 4. Discussion

CEA is a nonspecific tumor-associated antigen that is commonly expressed in gastrointestinal malignancies, and it has been widely used in clinical diagnosis and prognosis evaluation. Recent research has reported that CEA was correlated with TNM staging of CRC and showed a better estimation ability of metastasis and recurrence [18, 19]. In this study, we found that the level of CEA was significantly higher in CRC patients (*P* < 0.01), and the AUC was 0.75 (95% CI 0.71-0.79). At the threshold of 2.63, CEA had a moderate sensitivity (77.78%, 95% CI 0.72-0.83) and low specificity (64.51%, 95% CI 0.59-0.70). Therefore, there is a need for an improved tumor marker for CRC.

The inflammatory response is closely related to tumor development, and inflammatory factors initiate cell carcinogenesis and promote the migration and spread of tumor cells [20, 21], such as neutrophils, lymphocytes, platelets, and monocytes. Thus, NLR, d-NLR, PLR, and MLR that represent systematic inflammatory response are potential diagnostic markers for CRC. Recent studies reported that NLR had a good diagnostic value in CRC [22, 23], and d-NLR and PLR were correlated with the prognosis of CRC [24–26]. Our study found that NLR, d-NLR, PLR, and MLR can be used to distinguish patients with CRC from healthy volunteers. In addition, we used ROC curves and concluded that NLR, d-NLR, and PLR had good diagnostic value and PLR with the largest AUC area and higher sensitivity and specificity. Our results also showed that MLR was poorly diagnostic biomarker, for its AUC was <0.70, which is consistent with the result reported by Ying et al. [27]. However, this is contradictory to the result reported by Li et al. [8]. One reason for the discrepancy might be the different cutoff values used in this study, and the other reason was due to tumor specificity and underlying genetic and biological differences between distinct patient cohorts. Thus, we excluded MLR in the subsequent analysis. The correlation between inflammatory markers and clinicopathological features was analyzed, and we found that NLR, d-NLR, and PLR were only associated with TNM stage, and there was a low positive correlation between CEA and NLR and d-NLR.

The detection of single tumor markers has some degree of defects; thus, combined detection of multiple tumor markers can effectively improve the diagnosis of tumor [28, 29]. To further improve the diagnostic efficiency of CRC, we studied the combinations of CEA and PLR and of CEA and CNDNP in this study. The results showed that CEA and CNDNP have the highest combined diagnostic efficacy (AUC = 0.83, 95% CI 0.79-0.86) and could thus potentially serve as a valuable diagnostic indicator for CRC. Although combined detection of multiple markers can remedy the defects of single tests, and improve the sensitivity of diagnosis of CRC, it is not better to combine freely. For example, in our study, the sensitivity and specificity of the combined detection of NLR, d-NLR, and PLR were not significant compared with those of PLR and CEA alone. Therefore, we should combine clinical manifestations, pathological histology, and imaging to make a comprehensive diagnosis [4] and try to select indices with higher sensitivity and specificity to minimize the rate of misdiagnosis and improve the accuracy of diagnosis. However, all subjects in this study were from a single center, and there may be selection bias. Next, we will continue to increase the number of specimens, confirm the clinical trials of all ages and nationalities, and fully tap the clinical value of CNDNP.

In conclusion, combining inflammatory cell ratios with CEA can improve the diagnostic efficacy of CRC. The combination of CEA with CNDNP might be a useful indicator for CRC diagnosis and has better diagnostic value than other combined markers.

## Figures and Tables

**Table 1:** Clinicopathological features in the cancer and healthy groups.

Groups	Cancer group	Healthy group	*P*
*n*	324	216	<0.01
CEA (ng/mL)	14.08 ± 47.94	2.21 ± 2.09	<0.01
NLR	2.82 ± 1.92	1.94 ± 2.13	<0.01
d-NLR	2.01 ± 1.33	1.36 ± 0.43	<0.01
PLR	177.45 ± 89.75	135.99 ± 157.94	<0.01
MLR	0.30 ± 0.35	0.21 ± 0.16	<0.01

CEA: carcinoembryonic antigen; NLR: neutrophil-to-lymphocyte ratio; d-NLR:derived neutrophil-to-lymphocyte ratio; PLR: platelet-to-lymphocyte ratio; MLR: monocyte-to-lymphocyte ratio.

**Table 2:** Comparison of ROC curves of relevant inflammatory indices.

Detection index	Cutoff value	AUC	*95% CI*	Sensitivity (%)	95% CI	Specificity (%)	95% CI
NLR	2.10	0.74	0.70-0.78	78.70	0.73-0.84	58.33	0.53-0.64
d-NLR	1.73	0.71	0.67-0.75	83.33	0.78-0.88	50.31	0.45-0.56
PLR	146.71	0.75	0.72-0.79	84.72	0.79-0.89	57.72	0.52-0.63
MLR	0.22	0.68	0.64-0.72	74.07	0.68-0.80	54.94	0.49-0. 60

AUC: area under the curve; CI: confidential interval.

**Table 3:** Relationship between preoperative NLR, d-NLR, and PLR and clinicopathological features.

Variable	Patients (*n* = 324)	NLR			d-NLR			PLR		
		>2.10	≤2.10	*P*	>1.73	≤1.73	*P*	>146.71	≤146.71	*P*
Age (years)										
≥60	172	100	72	0.96	87	97	0.39	96	76	0.46
<60	152	88	64		79	73		91	61	
Sex										
Male	188	117	71	0.07	101	87	0.29	104	84	0.31
Female	136	71	65		65	71		83	53	
TNM stage										
*Ι* + II	218	137	81	0.01	99	119	<0.01	111	107	<0.01
III + IV	106	51	55		67	39		76	30	
Location										
Colon	168	96	72	0.74	98	70	0.12	93	75	0.37
Rectum	156	92	64		68	88		94	62	
Grade										
High	89	51	38	0.89	49	40	0.23	56	33	0.24
Moderate	123	70	53		67	56		64	59	
Low	112	67	45		50	62		67	45	

**Table 4:** Comparison of ROC curves of different combined detection schemes.

Joint indices	Cutoff value	AUC	95% CI	Sensitivity (%)	95% CI	Specificity (%)	95% CI	*P*
CEA	2.63	0.75	0.71-0.79	77.78	0.72-0.83	64.51	0.59-0.70	<0.01^a^
CEA+PLR	0.48	0.80	0.76-0.83	68.06	0.61-0.74	78.40	0.74-0.83	<0.05^b^
CNDNP	0.61	0.74	0.70-0.78	81.02	0.75-0.86	58.64	0.53-0.64	<0.01^c^
CEA+CNDNP	0.52	0.83	0.79-0.86	77.78	0.72-0.83	72.50	0.67-0.77	<0.01^d^

CNDNP: combination of NLR, d-NLR, and PLR. ^a^vs. CEA + PLR; ^b^vs. CNDNP; ^c^vs. CEA+ CNDNP; ^d^vs. CEA.

## Data Availability

The datasets used or analyzed during the current study are available from the corresponding author on reasonable request.
